# Neuropsychiatric manifestations in Cornelia de Lange syndrome

**DOI:** 10.1192/j.eurpsy.2023.1088

**Published:** 2023-07-19

**Authors:** F. Majdoub, A. Souissi, M. Trabelsi, A. Ziadi, N. Belguith, F. Maazoul, M. Guirat, I. Boujelbene, H. Kamoun, R. Mrad, S. Masmoudi, I. Ben Ayed

**Affiliations:** 1 Medical Genetics Department, University Hedi Chaker Hospital of Sfax, Tunisia., Medical school of Sfax, Tunisia; 2 Laboratory of Molecular and Cellular Screening Processes (LPCMC), Center of Biotechnology of Sfax, University of Sfax, Sfax; 3 Department of Hereditary and Congenital Disorders, Charles Nicolle Hospital, Tunis, Tunisia

## Abstract

**Introduction:**

Cornelia De Lange syndrome (CdLS) is a dominant and rare genetically heterogeneous syndrome. It is characterized by a large phenotypical spectrum going from a classical to a non-classical form affecting multiple organ systems including central nervous, locomotor, skin, gastrointestinal, immune and endocrine systems in association with specific dysmorphic features. Neuropsychiatric manifestations represent a hallmark of CdLS phenotype.

**Objectives:**

The aim of this study is to describe the neuropsychiatric features of Cornelia De Lange syndrome.

**Methods:**

This is a descriptive and retrospective study compromising unrelated Tunisian patients diagnosed clinically and genetically with CdLS during the period between 2002-2021. Each patient underwent a comprehensive clinical evaluation. In this study, we focused on neuropsychiatric and behavioural phenotype specifying intellectual disability(ID), language delay (LD), autism spectrum disorder (ASD), hyperactivity, aggressivity, specific learning disorder(SLD), sleep problems, compulsive behaviours and social anxiety disorders during adolescence.

**Results:**

A total of nine patients were included in this study. ID was present in all the evaluated patients with different level of severity evolving from mild (8/9) to severe (1/9). LD in absent of hearing problems was detected in two patients. Hyperactivity was found in three patients. Aggressivity was discovered in one patient in a form of self-injurious behaviour in one patient and hetero-aggressivity in another. None of our patients was diagnosed with ASD. Sleep problems such as frequent night-time awakenings were observed in one patient. All patients at age of schooling presented different levels of SLD. None of our patients was diagnosed with anxiety or compulsive behaviours.

**Conclusions:**

Our results support the implication of behavioural and psychiatric features in CdLS phenotype. All of symptoms described in the literature were present in our patients. Further evaluation of our patients during their life is important to reveal age-related features such as anxiety or compulsive behaviours. These features can be used to inform specific psychiatric assistance for family psychoeducation, psycho-social interventions, and cognitive-behavioural education treatment approaches in individuals with CdLS.

**Disclosure of Interest:**

None Declared

